# Progress on the Chemical Constituents Derived from Glucosinolates in Maca (*Lepidium meyenii*)

**DOI:** 10.1007/s13659-018-0185-7

**Published:** 2018-08-27

**Authors:** Yan-Jie Huang, Xing-Rong Peng, Ming-Hua Qiu

**Affiliations:** 1 State Key Laboratory of Phytochemistry and Plant Resources in West China, Kunming Institute of Botany, Chinese Academy of Sciences, Kunming, 650201 Yunnan China; 2 Yunnan Key Laboratory of Natural Medicinal Chemistry, Kunming Institute of Botany, Chinese Academy of Sciences, Kunming, 650201 Yunnan China; 3 University of Chinese Academy of Sciences, Beijing, 100049 People’s Republic of China

**Keywords:** Maca (*Lepidium meyenii* Walp.), Chemical constituents, Thiohydantoins, Biosynthetic pathway

## Abstract

**Abstract:**

Maca (*Lepidium meyenii* Walp.), a famous food supplement, has drawn an unprecedented international interest over the last two decades. It was assumed that glucosinolates, macamides, macaenes, and alkaloids are the main bioactive components of Maca before. Recently, a series of novel thiohydantoins which generally exhibit a variety of activities have been isolated from Maca. This review focuses on the progress on the main bioactive components of Maca and their biosynthetic pathway, which indicates that macamides, thiohydantoins, and some alkaloids may originate from glucosinolates. Interestingly, thiohydantoins from Maca are the first type of thiohydantoin derivatives to be found from a natural source and may contribute to some significant effects of Maca.

**Graphical Abstract:**

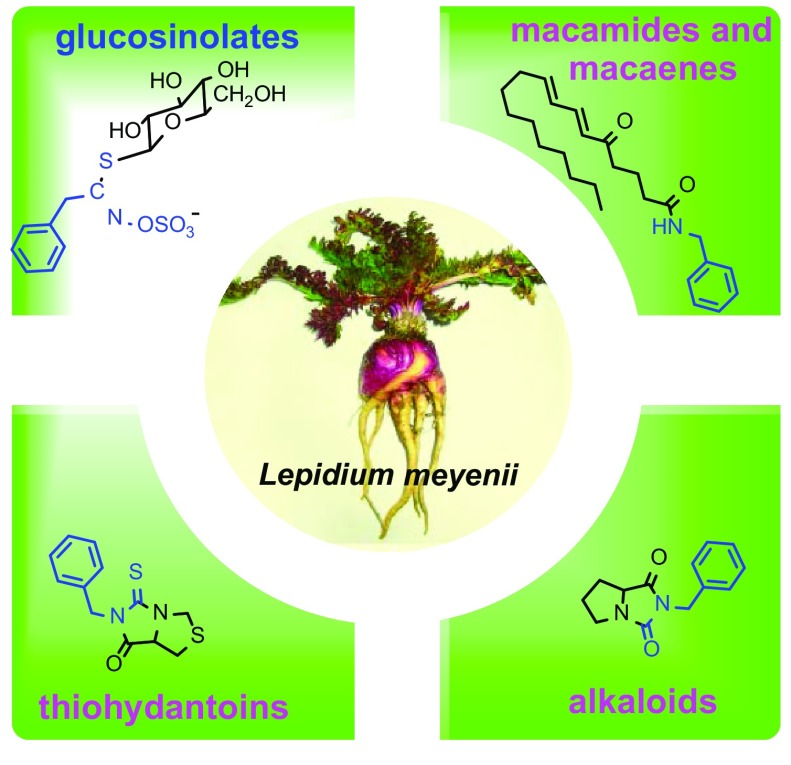

## Introduction

Maca (*Lepidium meyenii* Walp.), a species of the family Brassicaceae, has been cultivated both as a crop and medicinal plant for 1300–2000 years in Peru. The plant was used by indigenous Andean people for its invigorating effects, enhancing fertility in humans and cattle, and other medicinal properties such as curing respiratory disorders and anaemia [1].

According to the attributed health claims of increasing vitality and longevity, enhancing fertility and libido, suppressing malnutrition, Maca has drawn an unprecedented international interest through a marketing promotion as the “Andean Viagra” and “Peruvian Ginseng” via the media and internet from the 1990s. After Maca was approved by The Ministry of Health of the People’s Republic of China in 2002, it has been domesticated and cultivated in many zones, such as Yunnan, Xinjiang, and Tibet [2]. In China, Maca was firstly cultivated in Lijiang, Yunnan Province. As of today, the area of cultivation in Lijiang has reached approximately 9333 ha, which has become the biggest region to produce Maca [3].

Secondary metabolites in Maca have been categorized into several groups: glucosinolates, macamides, macaenes, alkaloids, sterols, and fatty acids. Over the last two decades, a succession of reviews have addressed the chemical constituents and biological activities of Maca, which indicate that glucosinolates, macamides, macaenes, and alkaloids are the main bioactive components [1, 3–5]. Recently, a series of novel thiohydantoins were isolated from Maca. These compounds may be another kind of bioactive components since compounds with the thiohydantoin moiety generally showed a variety of activities, such as antiparasitic, antituberculosis, and anticancer activities [6–9]. This review provided a comprehensive survey of the chemical structures of the main bioactive components of Maca based on available information from Web of Science and SciFinder Scholar database, and also elucidated the relationships among these four types of compounds on the basis of their biosynthetic pathway.

## Chemical Constituents with Bioactivity in Maca

There are several kinds of chemical constituents with bioactivity in Maca, including glucosinolates, macamides, macaenes, thiohydantoins, and alkaloids. These components are so distinctive and interesting that it inspires us to explore their biological pathway. This review provides a critical survey of the chemical structures from Maca, all of which are determined by the original literatures.

### Glucosinolates

Glucosinolates are *β*-thioglucoside *N*-hydroxysulfates, also known as (*Z*)-*N*-hydroximinosulfate esters, with a side chain (R) and a sulfur-linked *β*-d-glucopyranose moiety. At least 120 different glucosinolates have been identified from sixteen families of dicotyledonous angiosperms [10]. In Brassicaceae family, hundreds of species have been investigated and all of them are able to yield glucosinolates. The capacity to biosynthesize glucosinolates has been used as a taxonomic marker to support evolution-based classification schemes [10]. Different species contain different types of glucosinolates, but each plant generally contains six high-content and several trace amounts of glucosinolates.

**Fig. 1:**
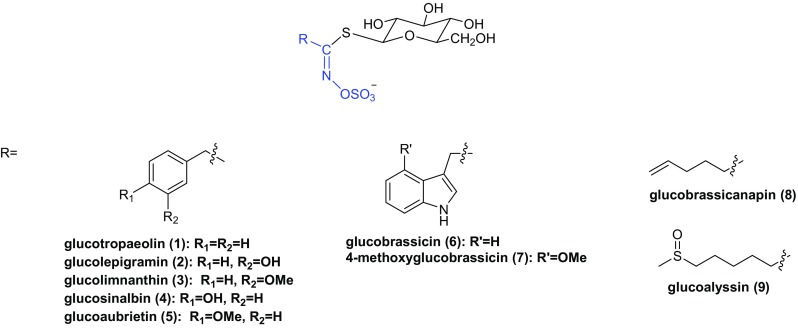
Structures of glucosinolates found in Maca

Glucosinolates are also a kind of secondary metabolites in Maca, which are considered to be responsible for the distinctive, pungent flavor of Maca [11]. Nine glucosinolates (**1–9**) have been found in Maca (Fig. 1), among which the aromatic glucosinolates represent a 99% average of the total glucosinolates content [5]. At harvest, glucotropaeolin (**1**) is the most abundant compound, representing around 80% of the total glucosinolates, followed by glucolimnanthin (**3**) [12].

**Scheme 1:**
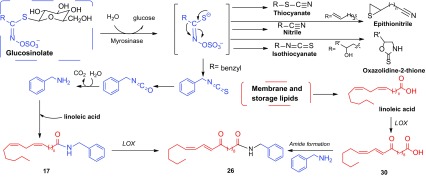
Possible reaction products of glucosinolate hydrolysis and hypothetical biosynthetic pathway of macamides. It is assumed that macamides are derived from two main substrates: free fatty acids and benzylamine. These two main substrates arise from the hydrolysis of membrane and storage lipids, and glucosinolates, respectively. The oxidation of free fatty acids bring macaenes out

Glucosinolates are stable water-soluble precursors of isothiocyanates and located in vacuoles. When the plant tissues are damaged, myrosinase which is stored in myrosinase grains of the myrosin cells will be released and combined with glucosinolates to produce biologically active compounds, such as nitriles, thiocyanates and isothiocyanates, epithionitriles, and oxazolidine-2-thiones (Scheme 1) [10, 13]. Glucosinolates and their metabolites are known for their cancer chemoprotective activity, primarily as inducers of Phase 2 enzymes with potential antiproliferative, apoptosis-promoting, redox regulatory activities [10]. The effects of glucotropaeolin (**1**) on endurance capacity in mice have been tested, suggesting that glucotropaeolin (**1**) is able to enhance swimming endurance by increasing the utilization of fatty acid as an energy source [14]. The glucosinolate-myrosinase system is also a chemical defense system for plants, which is known as the “mustard oil bomb”, because the most common class of the hydrolysis products, isothiocyanates (mustard oils), is widely believed to be highly toxic to insect herbivores [15].

### Macamides and Macaenes

Macamides and macaenes are assumed to be the characteristic marker compounds of Maca as they haven’t been found in any other plants. Macamides are considered to be formed from the combination of a benzylamine and a fatty acid moiety [5]. HPLC analyses show that macamides are absent in fresh undamaged Maca tissues while the hypocotyls dried by traditional Andean post-harvest practices or by an industrial oven contain up to 800 µg g^−1^ dry weight of macamides [16].

**Fig. 2:**
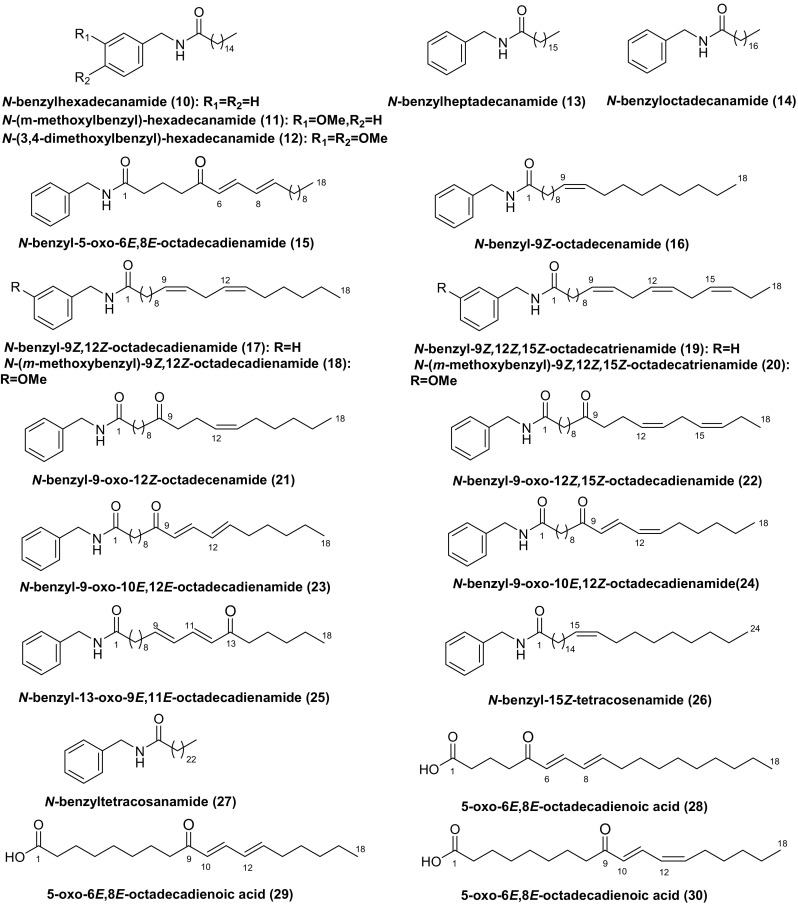
Structures of macamides and macaenes found in Maca

Thus far, eighteen macamides (**10–27**) and three macaenes (**28–30**) have been found in Maca (Fig. 2), among which compounds **10–12**, **15**, **17**, **19–30** were isolated as new compounds [17–21], compounds **13**, **14**, **16**, **18** were identified by HPLC–UV-MS/MS, UHPLC-MS/MS or HPLC–UV [16, 21–23]. Six major macamides (**10**, **11**, **16–19**) have been quantified in the ethanol extracts of Maca. The results show that *N*-benzylhexadecanamide (**10**) is the most abundant compound in Maca from Peru, while *N*-benzyl-9*Z*,12*Z*-octadecadienamide (**17**) is the richest compound in the Yunnan Maca and is the second highest abundant compound in Maca from Peru [22].

Macamides, the symbolic compounds of Maca, are endowed with a reasonable hypothetical biosynthetic pathway. Chen et al. assume that the biosynthetic pathway of macamides include 3 steps: firstly, glucosinolates are hydrolyzed by myrosinase to yield benzyl isothiocyanates, followed by being converted to benzylamines; secondly, membrane and storage lipids are hydrolyzed into free fatty acids; lastly, macamides are formed through the combination of one molecule each of benzylamine and fatty acid moiety [24, 25]. According to the fact that fresh hypocotyls of Maca showed high level of benzyl glucosinolate but undetectable levels of free fatty acids, benzyl amine and macamids, Esparza et al. proposed a biosynthetic scheme for macamides (Scheme 1) [16].

Macamides could easily cross the intestinal wall and the blood–brain barrier since they are neutral lipids. A series of macamides and their synthetic analogues were evaluated for their fatty acid amide hydrolase (FAAH) inhibitory activity. The results showed that macamides exhibited moderate FAAH inhibitory activity [26]. Moreover, the most active macamide, *N*-(*m*-methoxybenzyl)-9*Z*,12*Z*-octadecadienamide (**18**), displayed significant time-dependent, mixed non-competitive FAAH inhibitory activity, which suggested that this compond could have potential neuroprotective, analgesic, and anti-inflammatory activities [27]. Furthermore, *N*-benzyl-9*Z*,12*Z*-octadecadienamide (**17**) showed selective binding affinities for the cannabinoid CB_1_ receptor and a potent inhibition of anandamide cellular uptake, which uncovers a direct and indirect cannabimimetic action of Maca [19].

### Thiohydantoins

**Fig. 3:**
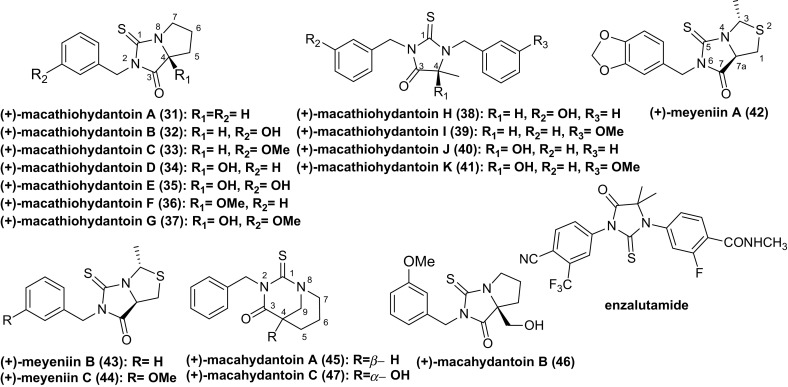
Structures of thiohydantoins found in Maca and enzalutamide

**Scheme 2:**
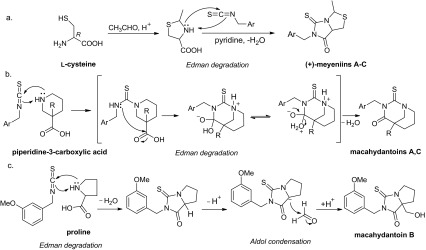
Hypothetical biosynthetic pathway of some thiohydantoins from Maca

Recently, seventeen thiohydantoins (**31–47**) have been isolated from Maca (Fig. 3). All of them are racemates [28–31]. Zhou et al. postulated the biosynthetic pathway of (+)-meyeniins A**–**C (**42–44**), involving the construction of the 2-methylthiazolidine ring by condensation reaction between acetaldehyde and l-cysteine, followed by Edman degradation reaction between 2-methylthiazolidine-4-carboxylic acid and related isothiocyanate (Scheme 2a) [29]. Considering the fact that natural isothiocyanates occur commonly in cruciferous plants, Zhou et al. hypothesized that macahydantoins A and C (**45**, **47**) could be more easily constructed by a simple Edman degradation reaction between related isothiocyanate and piperidine-3-carboxylic acid (Scheme 2b) [31]. Similarly, macahydantoin B (**46**) might be formed by a combination of Edman degradation and Aldol condensation from proline and related isothiocyanates (Scheme 2c). It’s noted that the biomimetic synthesis of meyeniins A**–**C, macahydantoins A and B were efficiently accomplished based on their biosynthetic hypothesis.

**Fig. 4:**
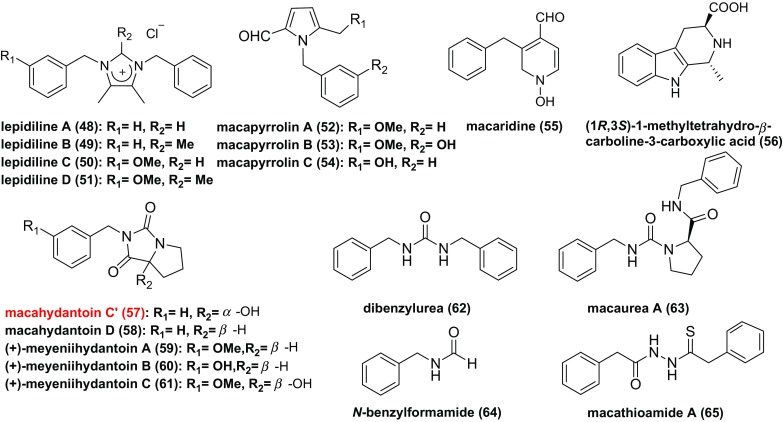
Structures of alkaloids found in Maca

The exocyclic, double bonded sulfur atom in thiohydantoins offers a particularly high density of interaction sites for polar interactions and hydrogen bonds, which endows thiohydantoins with the ability to form interactions with a variety of biological targets. And this is particularly pronounced if the substituted functions are aromatic, such as benzyl thiohydantoins [32]. Thus, compounds with the thiohydantoin moiety present a variety of applications, such as hypolipidemic [33], anticarcinogenie [34], antimutagenic [35], antithyroidal activities [36]. Recently, a high throughput screening and extensive structure–activity relationship optimization based on antiparasitic activity led to two highly potent compounds with a *Trypanosoma brucei* EC_50_ of 3 and 2 nM, respectively [6]. And a series of novel functionalized polysubstituted thiohydantoin-pyrrolidine derivatives were prepared for their antituberculosis activity, and these compounds showed moderate activity [7]. Moreover, a novel skeleton of indoline thiohydantoins was synthesized based on enzalutamide (Fig. 4), a newly approved nonsteroidal androgen receptor antagonists to treat prostate cancer. Several compounds showed good biological profiles in androgen receptor binding and higher selective toxicity than enzalutamide toward androgen receptor cells [8, 9].

However, seventeen thiohydantoins isolated from *L. meyenii* were tested for their cytotoxic activities against a panel of human cancer cell lines. Only (+)-meyeniin A showed moderately selective cytotoxic activity against the HL-60, A549, and MCF-7 human cell lines. Some compounds were tested for their acetylcholinesterase inhibitory activity, antimicrobial, and antifungal activity, but none of them showed significant activity [28–31].

### Alkaloids

**Scheme 3:**
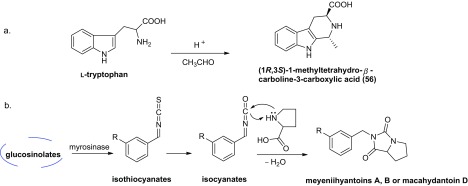
Hypothetical biosynthetic pathway of some alkaloids from Maca

Eighteen alkaloids (**48–65**) have been isolated from Maca (Fig. 4), including four imidazole alkaloids (**48–51**), three pyrrole alkaloids (**52–54**), five hydantoin alkaloids (**57–61**), two urea alkaloids (**62**, **63**) and four other alkaloids (**34**, **35**) [17, 37–43]. Among them, meyeniihydantoins A**–**C (**59–61**) are racemates. (1*R*,3*S*)-1-methyltetrahydro-*β*-carboline-3-carboxylic acid (**56**), which arises from a Pictet–Spengler condensation between l-tryptophan and aldehydes (Scheme 3a), generally occurs in foods and its formation is temperature and pH dependent [44]. Notely, there are double compounds (**47**, **57**) which are named macahydantoin C, herein compound **57** is named macahydantoin C’ to distinguish them [31, 41].

Although no literature has given the hypothetical biosynthetic pathway of hydantoins from Maca, we proposed one following the hypothetical biosynthetic pathway of macamides and thiohydantoins (Schemes 1, 2c). Thus, meyeniihydantoins A, B and macahydantoin D may be constructed by Edman degradation between related isocyanates and proline (Scheme 3b).

Among these alkaloids, some compounds (**48**, **49**, **52–54**, **59–61**) were evaluated against a panel of human cancer cell lines, among which lepidiline A (**48**) showed weak activity against the FDIGROV cell line, and lepidiline B (**49**) presented cytotoxic activity against the UMUC3, PACA2, MDA231, and FDIGROV cell lines [37]. However, macapyrrolins A**–**C (**52–54**) and meyeniihydantoins A**–C** (**59–61**) showed no significant cytotoxic activities at concentrations up to 40 µM [39]. Besides, tetrahydro-*β*-carbolines were reported to exert many activities on the central nervous system where they could function as neuromodulators [40].

## Conclusion

This review focuses on progress of four types of chemical components from Maca, including glucosinolates, macamides, macaenes, thiohydantoins, and alkaloids, which are the main bioactive components of Maca. Their biosynthetic pathway have been reviewed in this article for the first time, which reveals that macamides, thiohydantoins and some alkaloids may originate from related glucosinolates. We proposed the biosynthetic pathway of hydantoins, although no literature has reported it. All of these efforts make the chemical components of Maca more vivid and provide us a novel idea to investigate this plant.
